# Evaluating How Smartphone Contact Tracing Technology Can Reduce the Spread of Infectious Diseases: The Case of COVID-19

**DOI:** 10.1109/ACCESS.2020.2998042

**Published:** 2020-05-27

**Authors:** Enrique Hernández-Orallo, Pietro Manzoni, Carlos Tavares Calafate, Juan-Carlos Cano

**Affiliations:** Computer Engineering DepartmentUniversitat Politècnica de València 16774 46022ValenciaSpain

**Keywords:** Mobile computing, opportunistic networking, epidemic models, social networks, digital epidemiology

## Abstract

Detecting and controlling the diffusion of infectious diseases such as COVID-19 is crucial to managing epidemics. One common measure taken to contain or reduce diffusion is to detect infected individuals and trace their prior contacts so as to then selectively isolate any individuals likely to have been infected. These prior contacts can be traced using mobile devices such as smartphones or smartwatches, which can continuously collect the location and contacts of their owners by using their embedded localisation and communications technologies, such as GPS, Cellular networks, Wi-Fi, and Bluetooth. This paper evaluates the effectiveness of these technologies and determines the impact of contact tracing precision on the spread and control of infectious diseases. To this end, we have created an epidemic model that we used to evaluate the efficiency and cost (number of people quarantined) of the measures to be taken, depending on the smartphone contact tracing technologies used. Our results show that in order to be effective for the COVID-19 disease, the contact tracing technology must be precise, contacts must be traced quickly, and a significant percentage of the population must use the smartphone contact tracing application. These strict requirements make smartphone-based contact tracing rather ineffective at containing the spread of the infection during the first outbreak of the virus. However, considering a second wave, where a portion of the population will have gained immunity, or in combination with some other more lenient measures, smartphone-based contact tracing could be extremely useful.

## I. Introduction

The recent COVID-19 pandemic poses a major threat to our society and way of life. To date, hundreds of thousands of people have died, and millions infected. Most countries have taken severe measures, such as quarantines, lockdowns and social distancing, which continue to impact the population dramatically. In addition, the impact on the world economy is still hard to predict.

Countries and regions have tried tackling the COVID-19 outbreak in different ways to varying results. Countries such as South Korea and Israel took action quickly just as the first infectious cases appeared. They started checking anyone with possible COVID-19 symptoms and, when a case of infection was detected, their contacts were traced in order to detect new cases. The use of mobility traces (mobile communication and GPS positions) was instrumental to accomplishing this task ([1]), and the epidemic was controlled without taking further severe measures. Other countries, however, despite taking some measures after the initial outbreak such as quarantining infected people or checking their direct contacts only, did not manage to control the outbreak and eventually had to take draconian measures, such as lockdowns and social distancing.

Recent studies based on mathematical models have shown that asymptomatic individuals have caused around 80% of infections ([2]). Detecting COVID-19 is especially challenging because most infected individuals only have mild or even no symptoms. An important lesson can be learnt from this experience: early detection and early response is key to containing the initial outbreak. Therefore, fast and accurate contact tracing is needed to aid in controlling epidemic diseases. Contact tracing is a more selective isolation measure targeting the subset of the population most likely to have the infection, that is, individuals that have been in contact with infected and detected individuals [3]. However, as has been seen with COVID-19, if the average number of neighbours and the basic reproductive rate are high, contact tracing has to be far more efficient and rapid.

The widespread presence of mobile phones and increased availability of data and computing power can provide a ubiquitous way of tracking infectious diseases. This is a new approach to dealing with epidemics, known as *digital epidemiology*, which uses data generated outside the public health system [4]. To be specific, smartphone contact tracing entails using smartphones to collect the location and contact details of their owners, so that when people do get infected, their mobile can be used to trace their prior contacts so as to locate anyone else who might have also potentially been infected. These people could then be isolated (quarantined) themselves, thus limiting the spread of infection. Recently, and with the urgent aim of dealing with COVID-19, several contact tracing mobile Apps have appeared, such as the Singapore Government’s TraceTogether [5], Europe’s PEPP-PT [6], and MIT’s SafePath [7]. Lastly, Google and Apple teamed up in April 2020 to develop and integrate into their mobile operating systems what seems to be a definitive solution for contact tracing and whose only potential Achilles’ heel lies in issues of privacy.

The technology of these mobile apps is based on the results of several years of research in Mobile Computing, and particularly Opportunistic Networking (OppNet). OppNet [8] is based on the *opportunity* of exchanging messages between nearby devices when some type of direct and localised communication link is established (e.g., through a Bluetooth or WiFi direct channel). In some way, their behaviour and dynamics are similar to an epidemic spread of messages. In fact, many of the models used to evaluate these networks are an adaptation of well-known epidemic population models [9]. The study of mobility models and social behaviour was also key to evaluating the spread of information [10]. We believe that all this research background can help in coping with the spread of infectious diseases. In particular, we have not only studied the dynamics of message spreading and human mobility but have also developed several mathematical models and simulations tools [11]–[14] in this field. While the main goal of these previous research efforts was to improve the spread of information, our primary goal now is to reduce and halt the spread of infectious diseases.

In this paper, we focus on evaluating how smartphone contact tracing impacts the control and spread of the COVID-19 disease. We first evaluate and compare several contact tracing technologies and the relative methods for obtaining contacts. We then use temporal network graphs to characterise both the temporal contacts and the resolution and accuracy of the different tracing technologies studied. On the basis of these temporal graphs, we introduce a stochastic epidemic model that considers the individual contacts and the tracing technology. This stochastic model is later transformed into a deterministic model using parameters obtained from the stochastic models, thus allowing contact tracing to be evaluated for large populations in a fast and accurate way. On the basis of these models, we evaluate several possible scenarios for smartphone-based contact tracing, including a real mobility scenario based on real traces.

We believe this paper contributes significantly to a better understanding of the potentials and pitfalls of mobile contact tracing applications, particularly when applied to such extreme scenarios as COVID-19. Some of the most important outcomes are:

• Rapid contact tracing is extremely important for quickly isolating potentially infected individuals, particularly for a disease such as COVID-19, with a high reproduction rate and low detection rate. This result is in agreement with some recent results [15], [16], although our methodology differs substantially from the one used in these previous papers.
• Smartphone contact tracing precision mainly impacts the number of quarantined persons, since it allows for greater selectivity when quarantining citizens, thereby reducing the personal inconveniences and economical costs of these drastic measures. However, this accuracy has no significant impact on reducing the final number of infected individuals.
• Smartphone contact tracing, when adopted during a first outbreak, can only be effective when a fast and high-precision contact tracing technology is used, and when a significant proportion (more than 80%) of the population uses the smartphone application. These harsh requirements make it unlikely to be a feasible solution on its own.
• Fortunately, for future outbreaks, and under the condition that at least 20% of individuals gain immunisation or that the reproductive rate gets reduced by some other more lenient social distancing measures, smartphone contact tracing can be very effective, even when only a portion of the population is willing to use it (less than 60%).

The outline of this paper is as follows: an overview of related works is presented in Section 2. Section 3 introduces the mobile tracing technologies and contact networks that are used in the stochastic and deterministic models developed in Section 4. Section 5 is devoted to evaluating these models and their precision, and Section 6 evaluates the efficiency of the quarantine measures and tracing technologies. Finally, Section 7 details the main conclusions and future work.

## II. Related Work

Monitoring and controlling emerging infectious diseases is vital to public health. Through the use of new technologies such as internet-based surveillance, infectious disease modelling, remote sensing, telecommunications and mobile phones, these infectious diseases can be predicted, prevented and controlled [17]. This new approach for dealing with epidemics is usually categorised with a newly coined term: *digital epidemiology* [4].

Several works have evaluated the characterisation of human mobility patterns by using Call Detail Records (CDRs) for modelling and evaluating epidemic diseases [18]–[20]. Particularly, in [18], the authors explored the opportunity of using proxies of individual mobility to describe commuting flows and predict the diffusion of an influenza-like-illness epidemic. However, depending on the human mobility data source used, their predictive accuracy with regard to epidemic invasion timing and propagation patterns differed.

Another method for detecting and tracing contacts uses wireless sensor network technologies, such as Bluetooth or ZigBee. One of the first experiments using MOTES was performed by Salathé *et al.* [21]. They obtained high-resolution data of interpersonal contacts on one typical day at an American high school, which made it possible to reconstruct the relevant social network from an infectious disease transmission perspective. The paper also includes an SEIR (Susceptible, Exposed, Infectious, Recovered) model for evaluating disease diffusion and the impact of measures such as vaccination.

Mastrandrea and Barrat [22] also used wearable sensors to capture contacts between students and compared the results with contacts obtained from personal diaries. Furthermore, the authors compare how an epidemic disease was spread using two different contact networks (from sensors and diaries), which showed a notable difference in their dynamics.

Recent years have seen increasing interest in evaluating the efficiency and impact of contact tracing in epidemics. Contact tracing is a very useful measure focusing primarily on potential next-generation cases. Contact tracing has been proved to be a highly successful strategy when the number of infectious cases is low, or at the early stages of an outbreak, or especially when the disease may be asymptomatic (but still infectious), as it provides the only means by which such individuals can be easily identified [3]. Other studies evaluate the main factor in making an outbreak controllable [23], [24].

There are two main approaches for modelling contact tracing [25]: *Population-based modelling* is a top-down approach depicting disease dynamics on a system level that is typically used for analysing research matters from a macroscopic perspective; *Agent-based modelling* is a bottom-up approach dealing with each individual as an agent, each with their own movements and infection states, and is commonly used to evaluate heterogeneous and adaptive behaviours. In general, the latter method is more realistic, though it can be computationally demanding. Some papers, such as [26], [27], introduce a detailed stochastic model, which is reduced to a deterministic approach for obtaining the most fundamental dynamic of the epidemics and associated measures.

Most of these previous models only deal with generic networks. However, in order to evaluate the precision of trace contact models, we need to consider the network of contacts. For example, Huerta and Tsimring [27] introduced a stochastic model to evaluate the effect of contact tracing and random tracing as a part of the epidemic control strategy in complex networks. The paper shows that by tracing contacts, a major outbreak can be significantly reduced or even eliminated at low additional cost. This same stochastic model is also used by Farrahi *et al.* [28]. In this paper, the authors evaluate how communication traces can be obtained using mobile phones to estimate physical contacts, and thus help in selectively quarantining individuals. The results showed that contact tracing is only efficient at the beginning of the outbreak due to the rapidly increasing costs as the epidemic evolves. One of the main drawbacks of the stochastic model used in these two papers is its simplicity. For example, the model does not consider the possible quarantine of non-infected individuals or detected infected ones, as does the determinist model introduced by Keeling and Rohani [29]. Another approach is to model the spread of infectious diseases by considering the dynamic of the nodes using a temporal graph, as introduced by Yang *et al.* [30].

Some recent studies specifically deal with the COVID-19 pandemic. Ferretti *et al.* [15] claim that isolation and contact tracing as currently practised is not preventing the COVID-19 epidemic; this is mostly due to the high number of asymptomatic infected individuals that remain undetected, which contributes to the spread. Thus, they propose using mobile apps to trace the previous contacts, showing mathematically that epidemics can be contained even when not all the population uses the application (although the required portion is significantly high). Hellewell *et al.* [16] draw a similar conclusion through a simulated model. That is, in most scenarios, highly effective contact tracing and case isolation are enough to control a new outbreak of COVID-19 within 3 months, even when only 79% of the contacts are traced. Nevertheless, these conditions make smartphone-based contact tracing far from being a realistic solution.

One of the first attempts at using mobile phones to estimate contacts was the *FluPhone* application developed at Cambridge University [31]. That application used Bluetooth and other wireless signals as a proxy for estimating physical contacts and asked users to report flu-like symptoms in order to evaluate the risk of infections. For COVID-19, the Singapore Government developed and released the *TraceTogether* mobile App for tracing, which also relied upon Bluetooth contacts and had already been used to control disease spread [5]. Other similar proposals, also focusing on privacy issues, are the *Pan-European Privacy-Preserving* Proximity Tracing (PEPP-PT) [6] and *SafePaths* [7], [32]. Finally, Google and Apple have teamed up to develop and integrate similar solutions into the iOS and Android operating systems. As they are integrated into the operating system, the proposed solution is more efficient, and more importantly, will be ubiquitous for users.

Collecting personal mobility information, however, even for health application purposes, poses specific challenges when it comes to upholding ethical principles and issues of privacy [33]. Ultimately, the analysis of mobility data can be justified if it can yield benefits to public health.

This paper differs in that it specifically evaluates how smartphone contact tracing technology can reduce the spread of infectious diseases and the associated cost of the quarantine measures. To evaluate these contact tracing technologies, it is necessary to consider contacts individually and on a temporal basis. Additionally, as an example, we use a real scenario to evaluate these contact tracing technologies.

## III. Contact Networks and Tracing

In this section, we model contacts obtained via smartphones by using contact network graphs. We first perform an evaluation of the different technologies that can be used to trace the location of a mobile phone. Then, we describe a network graph to model these contacts and how these previous contacts can be estimated. Finally, using a real mobility trace (NCCU trace), we describe how we obtained the different graphs that will be used in the epidemic models.

Traditionally, these contact graphs were usually obtained manually through personal interviews, during which the patient tried to remember prior contacts or any locations he/she had visited. This approach is widely used for some kinds of diseases (particularly sexually transmitted ones) where the contacts are clear and easy to remember. For most infectious diseases, however, personal interviews provide very poor tracing of prior contacts and locations.

### A. Mobile Tracing Technologies

Mobile contact tracing applications are based on the idea of detecting contacts by using some of the latest localisation and communication technologies. For this paper in particular, we evaluate the ability of four different technologies to obtain a network of contacts.^1^ From lower to higher resolution and precision, we have:

1. *Cell*. The mobile phone network is distributed over land areas called cells. Telecommunications providers can determine the rough location of connected phones depending on which cell the mobile phone is connected to. However, the precision of the obtained location can be very low since the area of any one cell can vary from hundreds to thousands of meters, making the process of determining contacts from these traces very inaccurate. On the other hand, a great advantage of this technology is that communication providers are already obtaining and storing this information (legally, they are obliged to do so in most countries), and so it could be used when necessary (meeting, naturally the legal requirements). Thus, the economic cost of using this technology can be considered negligible when compared to other technologies.
2. *Wi-Fi*. Using local communication facilities, such as WiFi, we can determine the identity (MAC Address) of the surrounding devices. Thus, mobile nodes can periodically scan and store information about all these surrounding devices, which can also include the Received Signal Strength (RSSI) for estimating distance.
3. *GPS*. Smartphones are equipped with GPS, which allows them to be used to trace the user location. The precision of this solution is about 10 to 15 meters outdoors, although indoors the precision is severely reduced, making this a significant restriction since most infectious contact takes place indoors.
4. *Bluetooth*. Similar to Wi-Fi, although more precise. For example, using Bluetooth we can obtain a trace of the contacted devices with a resolution of 1–2 meters, making it ideal for determining close personal contact, which are the most likely to transmit infectious diseases.

^1^We have focused our study on widely available current communication and localisation technologies that can be used for implementing real contact tracing applications (such as those under development for the COVID-19 disease). Technologies requiring additional infrastructure and services (i.e. BLE beacons) or those not supported by current mobile phones (such as LiFi or UWB) are not considered.

Recent solutions using Wi-Fi and Bluetooth contact tracing are based on mobile apps that exchange anonymous key codes when a possible nearby contact is detected. This process generates data with the possible user contact information that must comply with each country’s regulations on data protection and privacy. This has led to two different models for managing and storing these data: centralised and distributed. In the centralised model, the anonymised data is uploaded from the users’ mobile phone to centralised servers. This way, the Health authorities can check and manage contacts. The decentralised model, on the other hand, stores these contacts locally and allow the users to voluntary check (or not) if they have been in contact with people who may have been infected. This approach is the one taken by the solution developed by Apple and Google. Both models (centralised and decentralised) can comply with most data protection and privacy regulations, but the decentralised approach may offer users a higher degree of privacy. However, the centralised model might provide Health authorities greater control and information about the spread of infections, and it may be faster at detecting new potentially infected individuals because it would not depend on the willingness of users to check their status.

In both cases, these technologies would require deployment not only of the mobile App but also of the required centralised servers to store and check the contacts, so the economical cost might be significant.

### B. Characterising Contacts

One common way of representing the interactions between individuals is through the use of network graphs. These networks can be a very useful tool for understanding the transmission of infections in human populations [29], [34] where each individual is in contact with only a small proportion of the population. We consider a population of $N$ individuals (the nodes) whose contacts are defined as a temporal graph $\mathbf {G}(t)$, where a link between nodes $i$ and $j$ represents a contact between them at time $t$. In epidemic models, it is very common to use a day as the time unit, so contact graphs represent the contact between individuals in one day. That is, $\mathbf {G}_{ij}(t)$ is 1 if the pair $(i,j)$ of individuals are in contact on day $t$, and 0 otherwise. Usually, for infection applications, this graph is symmetric ($\mathbf {G}_{ij}(t)=\mathbf {G}_{ji}(t)$), meaning that the infection can be passed in both directions. The temporal degree $K_{i}(t)$ of an individual $i$ is the number of links (contacts) between this individual and the other individuals per unit time (days). From this temporal degree, we can obtain the average degree $k$ of a contact network over a given time $T$ as:

\begin{equation*} k = \frac {1}{N} \sum _{i=1}^{N} \left ({\frac {1}{T} \int _{0}^{T} K_{i}(t) dt }\right) \tag{1}\end{equation*}

To evaluate the diffusion of the infection, it is useful to obtain the number of contacts with individuals that can transmit the infection only, as:

\begin{equation*} K^{*}_{i}(t) = \sum _{j=1}^{N} \mathbf {G}_{ij}(t) I_{j}(t) \tag{2}\end{equation*}

where $I_{j}(t)$ is 1 if individual $j$ at time $t$ can infect others, and 0 otherwise. Note, that $I_{j}(t)$ does not consider infected individuals that are isolated since they cannot transmit the infection.

Using the contact network graph, we can also trace prior contacts when an infected individual is detected, i.e. trace back contacts in order to quarantine individuals who are more likely to have the infection. In this case, we consider all the contacts occurring in a given period $\Delta $ (the backward time window). The idea is to trace back only recent contacts, and this time window will depend on the dynamics of the disease (for example, the incubation time). Summing up, we want to check if an individual has had contact with at least one traced individual during the previous backward time. We therefore define a function $C_{i}(t,\Delta)$ that checks it, returning 1 if it is true, and 0 otherwise:

\begin{equation*} C_{i}(t,\Delta) = \begin{cases} 1,& \displaystyle \sum \nolimits _{j=1}^{N} \left ({\max _{\tau \in [t-\Delta, t]} \mathbf {G}_{ij}(\tau) }\right) D_{j}(t) > 0 \\ 0, & \text {otherwise} \end{cases} \tag{3}\end{equation*}

where $D_{j}(t)$ is 1 if individual $j$ at time $t$ is detected and being traced, and 0 otherwise.

It is important to remember that the previous network graph $\mathbf {G}(t)$ is the *real* contact network, that is, the one that reflects the real physical contacts and, therefore, the transmission of the disease. As when trying to model most real systems however, it is almost impossible to obtain this real network. For contact networks, in particular, it has been shown to be impossible to obtain the real close-encounter interactions of a population using wearable devices [35]. Therefore, by using some of the previous tracing methods, we can only estimate the *real* contact networks by obtaining a new graph $\widehat {\mathbf {G}}(t)$, which is an estimation of the real one.^2^ In other words, they are a *noisy* measure of the real network.^2^This is similar to the dual graph model used in [28], although we prefer here to use the dynamic system notation.

A method for measuring how noisy this estimation is was introduced in [28]. We extend this method to consider temporal networks. The method considers two main differences between the estimated and real networks: *(i) removed links*, when some of the real contacts may not have been captured using mobile traces, and so some links from the real graph do not appear in the estimated one; and *(ii) added links*, when smartphone contact tracing apps generate some incorrect contacts that do not occur in the real world, causing some links to be added to the estimated graph that do not appear in the real one. By making $L^{r}(t)$ the number of removed links at time $t$, and $L^{a}(t)$ the added ones, and considering that $K(t)=\sum _{i=1}^{N} K_{i}(t)$ is the full number of links of the real network graph $\mathbf {G}(t)$ (that is, the daily contacts), we can define the error of the estimated network at time $t$ as:

\begin{equation*} \varepsilon (t) = \frac {L^{a}(t)+L^{r}(t)}{K(t)+L^{a}(t)} \tag{4}\end{equation*}

which ranges from 0 (both networks have the same links) to 1 (the networks differ completely, not sharing any links). If we consider a measured interval from 0 to $T$, we can obtain the average error as:

\begin{equation*} \bar {\varepsilon } = \frac {1}{T} \int _{0}^{T} \varepsilon (t) dt \tag{5}\end{equation*}

which will provide a measure of the precision of the estimated contact trace.

### C. NCCU Trace Analysis

To evaluate the different mechanisms that go into obtaining a network of contacts, we have used the NCCU Trace, a real-life mobility trace obtained at the NCCU University campus [36]. This NCCU Trace was collected using an Android app installed on the smartphones of 115 students attending the National Chengchi University, Taiwan. The trace was recorded over a period of 15 days and contains the GPS data, Wi-Fi access points, and Bluetooth devices in proximity. Time is specified with a resolution of one second, and the position information is rounded to meters.

**Figure 1.**
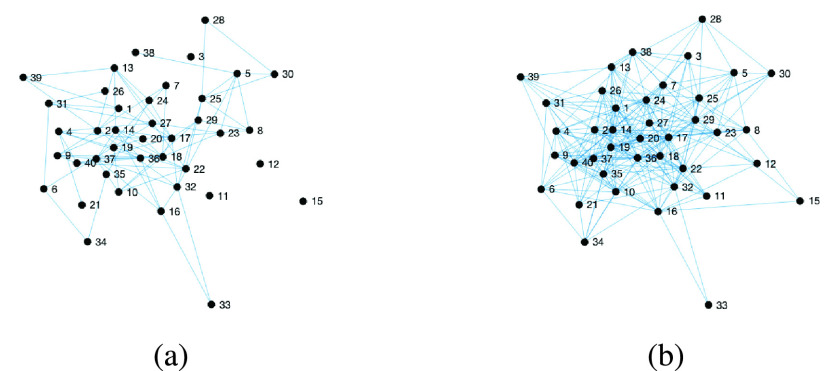
Contact network graph for the first 40 nodes during the first day. a) *Real* contacts; b) Bluetooth contacts.

It is important to remember that, in most real experiments, we cannot obtain the set of real physical contacts. Since we want to evaluate the precision of the different technologies (Mobile cells, GPS, Wi-Fi and Bluetooth), we consider that a real contact susceptible to producing an infection is one that is within the range of two meters and has a duration greater than 1 minute. Therefore, we processed the NCCU trace using these parameters to obtain the $\mathbf {G}(t)$ contact network graph. The result is a contact graph for 15 days with an average rank of 7.66 (i.e. the average number of contacts per day and individual). Figure 1 shows this contact graph for the first day and includes the first 40 individuals. For some experiments, 15 days may not be enough time to evaluate the spread of an infection. Thus, when required, the trace will be extended by repeating the previous weeks. It is assumed that mobility patterns are very similar over consecutive weeks. This fact is confirmed when analysing the contact time series, which show high auto-correlation for the same weekdays.

Since our goal is to evaluate different technologies for estimating contacts, we describe the different patterns and methods used to obtain these contacts. Note that most contact tracing applications only consider simple contact patterns, i.e. a contact occurs if two mobile devices are within a given distance for at least a predefined time. Thus, based on these technologies, we generated several estimated contact graphs $\widehat {\mathbf {G}}(t)$ as follows:

• *Cell*: The evaluated area of the campus is $3764\,\,m \times 3420\,\,m$, and we consider here a mobile cell network with four towers and a resolution of 200 meters. Contacts are obtained with this range in consideration.
• *Wi-Fi*: The range of varies depending on whether it is an open space or not. We consider a range of 25m indoors, and 100m outdoors. Thus, for generating the contacts, we evaluated whether the individuals are indoors (or outdoors) by checking that the actual GPS locations are inside (or not) a building area.
• *GPS*: In this case, we assume an outdoor accuracy of 5 meters, which increases to 25 meters indoors.
• *Bluetooth*: In this case, we consider a direct detection between mobile phones with a range of eight meters, in both indoor and outdoor locations.
• *Exact*: This would be the desired goal, to obtain a contact graph as close to the real physical contacts. It is included in our experiments for comparison purposes.

**Figure 2.**
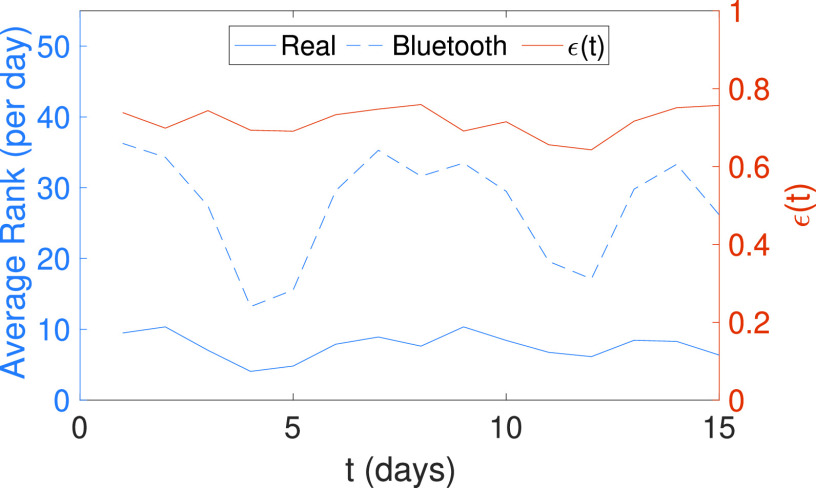
Temporal error and rank of the real and Bluetooth contact networks. The error is plotted against the right y-axis, and the average rank against the left y-axis.

In all cases, the minimal contact duration considered is one minute. From top to bottom, the average rank of the above list of estimated contact networks is as follows: { *Cell* 89.31, *Wi-Fi* 69.18, *GPS* 41.00, *Bluetooth* 27.48, *Exact* 7.66}, clearly showing that the range used is decisive when estimating the number of contacts. With more detail, we can see the Bluetooth contact graph in Figure 1b. When it is compared to the real graph, we clearly observe an increase in the number of edges (contacts) obtaining an error, as defined in Equation 5, of 0.73. Figure 2 shows the temporal error between the two graphs, with small variations between days (always in the range [0.65, 0.75]. This figure also shows the average rank per day, where we can see that the Bluetooth contacts are much higher than the real ones due to the higher range used to detect contacts.

## IV. Epidemic Model for Evaluating Contact Tracing Efficiency

In this section, we introduce our model for describing the individual contacts and tracing mechanism. We consider a SIR (Susceptible, Infected, Recovered) epidemic model with quarantine in a fixed population of $N$ individuals.^3^ We first create a stochastic model using the event-driven approach and considering each node independently. The contacts in this model are driven by the real contact network defined by $\mathbf {G}(t)$ and the estimated contact network $\widehat {\mathbf {G}(t)}$.^3^As a closed system, no natural births and deaths are considered. We consider that the time scale of the outbreak and the required tracing is small enough to allow for not taking into account this variation on the population.

When considering an average number of contacts and tracing intensity, this stochastic model can be transformed into a deterministic model (that is, a mean-field approximation), which results in an extended model of the contact tracing quarantine model introduced by Keeling and Rohani ([29], pp. 314-316). This continuous model is useful for evaluating the dynamics of the epidemic and the impact of the different quarantine methods in greater populations.

### A. The Stochastic Model

We consider a population of $N$ individuals whose *real* temporal contacts are determined by $\mathbf {G}(t)$. Individuals have six states: $S$, susceptible individual (not infected); $I$, infected individual; $R$, individual recovered from the infection; $Q_{I}$ infected individual that has been detected (or traced) and therefore quarantined; $Q_{S}$, a susceptible individual that is quarantined after being traced; and $Q_{T}$, an infected individual that has been detected and is being traced.

The model has for each individual eight possible events that imply a change of state that can occur at a defined rate.^4^ Formally speaking, the number of possible events is $8N$, since these events are for each individual. So, for example, an event from $S \rightarrow I$ for an individual will imply a change of state in this node. As a reference of these events, see Table 1, and also the notation in Table 2.^4^In other notations, this rate is multiplied by a small $\Delta t$ time interval in order to obtain the associated probability of the event.

**TABLE 1:** Events of the Stochastic Model

Event	Rate	Description
$S \rightarrow I $	$(1-q'\widehat {C_{i}}(t,\Delta)) b K^{*}_{i}(t)$	Infected individuals that are not traced.
$S \rightarrow Q_{S} $	$q'\widehat {C_{i}}(t,\Delta) (1-b K^{*}_{i}(t)) $	Non-infected individual traced and quarantined.
$S \rightarrow Q_{T} $	$q'\widehat {C_{i}}(t,\Delta) b K^{*}_{i}(t)$	Infected individuals that are being traced and go directly to the tracing state.
$I \rightarrow Q_{T} $	$\delta $	Detection of infected individuals, going to tracing.
$I \rightarrow R$	$\gamma $	Infected individual recovered from disease.
$Q_{S} \rightarrow S$	$\tau _{Q}$	End of quarantine for susceptible individual.
$Q_{I} \rightarrow R$	$\tau '_{Q}$	End of quarantine and infection.
$Q_{T} \rightarrow Q_{I}$	$\tau _{T}$	End of tracing, goes to the rest of their quarantine.

**TABLE 2:** Notation Table

Symbol	Definition
$N$	Population
$S,I,R$	Susceptible, Infected and Recovered individuals states
$Q_{S}$	Susceptibles in quarantine by tracing
$Q_{I}$	Infected detected and quarantined.
$Q_{T}$	Infected detected and being traced.
$Q_{a}$	Accumulated individuals being quarantined by tracing.
$R_{0}$	Basic reproductive ratio ($R_{0}=kb/\gamma $)
$K^{*}_{i}(t)$	Contacts per time unit with infected individuals, for node $i$.
$\widehat {C_{i}}(t,\Delta)$	Check if an individual has contacted with a traced one.
$k$	Average degree (i.e. average contacts per time unit and ind.).
$b$	Probability of transmitting the disease.
$\beta $	Transmission rate ($\beta =k \cdot b$)
$\gamma $	Recovery rate ($1/\gamma $ = days to recover)
$\delta $	Detection rate of infected individuals
$1/\tau _{Q}$	Average quarantine time
$1/\tau '_{Q}$	Average quarantine time minus $1/\tau _{T}$
$1/\tau _{T}$	Average tracing time
$q$, $q'$	Fraction of traced contacts quarantined (normalised $q'$)
$q_{i}$, $q_{s}$	Estimated fraction of traced contacts (deterministic model).
CFR	Case Fatality Rate.

The transmission rate, that is the rate at which the infection is transmitted from an infected individual to a susceptible one, can be obtained as the number of contacts at time $t$ with infected individuals $K^{*}_{i}(t)$, and the probability of transmitting the disease, $b$. The latter value depends on the type of disease. The infected individuals recover after $1/\gamma $ days, where $\gamma $ is the recovery rate. All these values are related to the basic reproductive ratio as $R_{0}=kb/\gamma $, where $k$ is the average degree of all individuals (see Equation 1). Thus, knowing some basic parameters of the disease ($R_{0}$, $\gamma $) and the contact network $k$, we can obtain $b$. $R_{0}$ is one of the most important figures in epidemiology and represents the expected number of cases directly generated by a single case. Therefore, when $R_{0} > 1$, the infection will start spreading in a population, but not if $R_{0} < 1$. Generally speaking, the larger the value of $R_{0}$, the harder it is to control the epidemic. Finally, the most common measure for the mortality rate is the Case Fatality Ratio (CFR), which is the number of deaths divided by the number of cases (infected). This value is not usually used in SIR epidemic models since their *behaviour* is the same as that of a recovered individual: they cannot infect other individuals. In Table 3 we can see several estimated values of these values for COVID-19.^5^^5^Note that these estimated values are drawn from very preliminary studies, and also depend on the location and country.

**TABLE 3:** Some Estimated Infectious Diseases Parameters (Time Unit Days). From [2], [15], [16]

Parameter	Estimated value
$R_{0}$	3 (1.5–6)
$\gamma $	1/15
$\beta $	0.52
$\tau _{Q}$	1/14
$\delta $	0.1 (0.05–0.1)

In our model, we suppose that when an infected individual is detected, he/she is immediately isolated. Then, his/her contact network is evaluated in order to find any individuals with a high probability of having been infected. These individuals are also quarantining. We consider two quarantine strategies in our model, namely the *Infected-Detected Quarantine* and the *Tracing Quarantine*.

The first strategy, the *Infected-Detected Quarantine* is the most common quarantine measure, where the infected and detected individuals are isolated. In our model, infected individuals are detected, traced and quarantined with a rate $\delta $ (event $I \rightarrow Q_{T}$), and stay in quarantine for an average time of $1/\tau _{Q}$ days. Before going into the final quarantine state $Q_{I}$, these individuals stay for a short time $1/\tau _{T}$ in state $Q_{T}$, where their previous contacts are traced. The time $1/\tau _{T}$ can model, for example, how long it takes to trace the contacts, allowing for a comparison of fast-tracing methods using mobiles phones versus traditional and slow-tracing methods. After this time $1/\tau _{T}$, they change to the $Q_{I}$ state, where they stay more time, which is the remaining average quarantine time: $1/\tau '_{T} = 1/\tau _{Q} - 1/\tau _{T}$. After that time, they recover and change to the $R$ state (event $Q_{I} \rightarrow R$). Also note that as with real epidemic spread, not all infected individuals are detected (for example, those having no symptoms or mild symptoms), and so they can still infect susceptible individuals. For those infected and not detected, they recover from the disease with a rate of $\gamma $ (event $I \rightarrow R$).

In the second quarantine strategy, the *Tracing Quarantine*, when infected individuals are detected (that is, when they are in state $Q_{T}$), their prior contacts in the susceptible state are traced using the estimated contact network $\widehat {\mathbf {G}}(t)$, and some of them are quarantined. Using Equation 3, an individual $i$ is a candidate for quarantine if $\widehat {C_{i}}(t,\Delta)$ is one, where $D_{j}(t)$ refers to the nodes that are in the $Q_{T}$ state. However, not all the traced individuals will be quarantined, so we define $q$ as the fraction of traced individuals being quarantined. For example, this value can reflect the number of individuals that use the mobile contact tracing app. In the case where the tracing time is greater than 1, the $q$ value must be normalised by the average tracing time, as $q'=q/(1/\tau _{T})=q\tau _{T}$ in order to distribute the tracing quarantine over the days. The idea is that if the tracing time is long (for example, by using interviews), it is precisely because it takes time to trace back the prior contacts, so the whole number of traced individuals during this tracing time is equally distributed over these days.

The rate at which all the susceptible individuals are traced is $q'\widehat {C_{i}}(t,\Delta)$. Furthermore, a portion of these individuals may have been infected during the period corresponding to one time unit (that is, during the day). Thus, infected people have a rate of $q'\widehat {C_{i}}(t,\Delta) b K^{*}_{i}(t)$, changing also to state $Q_{T}$, and consequently starting a new tracing. On the other hand, non-infected people change to class $Q_{S}$ with rate $q'\widehat {C_{i}}(t,\Delta) (1-bK^{*}_{i}(t))$. After being quarantined, individuals in $Q_{S}$ state go back to the susceptible state (event $Q_{S} \rightarrow S$), and individuals in $Q_{I}$ state change to the recovered state (event $Q_{I} \rightarrow R$).

Finally, an alternative and more draconian measure can be taken: a lockdown or *full quarantine*. That is, when the outbreak starts, the entire population is forced to isolate in their homes, and all public spaces are closed in order to drastically reduce the number of contacts as a way of stopping or slowing down the spread of the infection. This implies a change in the contact network, where the number of contacts is drastically reduced in order to reduce the reproductive ratio ($R_{0}$).

This event-driven stochastic model, as described in Table 1, can be solved using the Gillispie method [37]. This iterative method is based on estimating the time until the next event occurs by using the cumulative rates of all possible events. Considering $N$ individuals and an initial number of infected individuals ($I(0) < N$), we set $I(0)$ individuals in the *Infected* state, $R(0)$ individuals in the *Recovered* state,^6^ and the rest in the *Susceptible* state. This procedure is repeated for a given evaluation time $T$, or until the number of infected individuals is 0. We consider that these individuals have a physical contact network determined by graph $\mathbf {G}(t)$, and an estimated one defined by $\widehat {\mathbf {G}}(t)$. These graphs are obtained on a daily basis; thus, if we want to evaluate $T$ days, we will have $T$ different graphs, and the event times need to be rounded to a day.^6^For evaluating a first outbreak, the value of $R(0)$ will be 0. Nevertheless, for possible subsequent outbreaks, part of the population may be considered to have recovered and thus have immunisation, meaning that $R(0)>0$.

To evaluate the efficiency of the different isolation methods, we also compute the accumulated number of individuals quarantined using tracing methods ($Q_{a}$). To account for the individuals quarantined, $Q_{a}$ is initially set to zero, and it is incremented by one whenever an individual is quarantined by tracing (events $S \rightarrow Q_{T}$ and $S \rightarrow Q_{I}$ in Table 1). Note that sometimes, if the duration of the infection is long, individuals can be quarantined several times; that is, when they leave the quarantine they return to the susceptible class so they can be quarantined again.

The computational cost of this model depends on the number of individuals $N$ and on the average degree $k$ of the contact networks. The performed experiments show that its computational cost is exponential with $N$. Therefore, we need an alternative method when considering large populations, like the deterministic model we introduce in the next subsection.

### B. Deterministic Model

The stochastic model described above can be converted into a deterministic model, assuming a degree of homogeneity in the contact network. Thus, the precision of this model will depend not only on the homogeneity of the contacts but also on the number of nodes (individuals), where accuracy is greater when the number of nodes is high. In this deterministic model, the previously considered six states of an individual are now transformed into six classes, which represent the number of individuals in each state. Furthermore, to model the transmission, we use the number of average contacts ($k$) of the contact network.

To model the contact tracing methods, we use two different fractions of quarantined contacts, one for infected nodes, $q_{i}$ and another for susceptible ones, $q_{s}$. The reason for having these two fractions is to consider the effect that the real and estimated contact networks have on the tracing of contacts. The goal is to measure the precision of the technology used to retrieve the infected individuals when tracing. If the estimated contact network used for tracing has many more contacts than the real one (for example, when using the *Cell* one), it will trace more susceptible nodes than a more accurate one.

The first value, $q_{i}$ is obtained from the stochastic model as the average value of $q\widehat {C_{i}}(t,\Delta)$ for all $i$ using the real contact network as the estimated one (that is, considering perfect contact tracing). To obtain this value $q_{i}$, it is necessary to perform several realisations of the stochastic model, for example by using the Gillespie method. The second value, $q_{s}$ is obtained in a similar way but using the estimated contact network. In other words, the value $q_{i}$ determines the fraction of contacts with infected individuals being traced and potentially infected, and $q_{s}$ the equivalent for the ones not infected.^7^ In general, we can see that $q_{i} \leq q_{s} \leq q$, since the estimated contact network has more contacts than the real one, and both of them refer to the whole set of infected individuals, whereas $q$ only refers to a subset of the infected individuals being traced.^7^Note the difference between $q$ and $q_{i},q_{d}$: $q$ refers to the fraction of those individuals who have had contact with infected individuals being traced, whereas $q_{i},q_{d}$ refers to the fraction of those individuals who have recently had contact with someone infected (not only someone traced).

The transmission rate $\beta $, that is, the rate at which the infection is transmitted from *one* infected individual to *one* susceptible individual, is formed by the product of the number of contacts per time unit $k$, and the probability of transmitting the disease $b$; hence, newly infected individuals are generated with a rate $kbI\frac {S}{N}$. Regarding the tracing quarantine, the previously mentioned $\widehat {C_{i}}(t,\Delta)$ term will depend on the average rank of the estimated graph, considering only the fraction $q$ of contacted individuals. Taking into account the rates in Table 1 as well, the equations of the continuous model are as follows:

\begin{align*} S'=&-(1-q_{i})\frac {kbIS}{N} - q_{i}\frac {kbIS}{N} - q_{s}\frac {k(1-b)IS}{N} + \tau _{Q} Q_{S} \\ I'=&(1-q_{i})\frac {kbIS}{N} - \delta I - \gamma I \\ R'=&\gamma I + \tau _{Q} Q_{I} \\ Q_{S}'=&q_{s} \frac {k(1-b)IS}{N} - \tau _{Q} Q_{S} \\ Q_{I}'=&\tau _{T} Q_{T} -\tau '_{Q} Q_{I} \\ Q_{T}'=&\delta I + q_{i}\frac {kbIS}{N} -\tau _{T} Q_{T} \tag{6}\end{align*}

In all equations, we have omitted the time in the classes (e.g. for class $S$, $S'=dS(t)/dt$ and $S=S(t)$). Also, note that we have opted for not simplifying some expressions in order to clearly differentiate the different transition terms.

A key issue in epidemic control is reducing the number of infected individuals that remain undetected and who can contribute to the fast spread of the infection (this fact has been one of the main causes for the fast spread of COVID-19). This can be achieved by increasing the detection ratio, for example by increasing the number of tests, even for asymptomatic individuals, and also by increasing the traced individuals. From the previous model, we can obtain the conditions for an outbreak to be controlled, that is, when $I$ decreases. Thus, working out when the second equation in 6 is negative, we have:

\begin{equation*} (1-q_{i})\frac {kbS}{N} < \delta + \gamma \tag{7}\end{equation*}

This expression is similar to the one obtained by Keeling and Rohani [29] (pp. 315-316). Considering that $R_{0}=kb/\gamma $, we can obtain the threshold for an epidemic outbreak depending on the basic reproductive ratio $R_{0}$ and the proportion of susceptible people ($S/N$). Note that this expression depends on $q_{i}$ and not on $q_{d}$. This makes sense since only the detection and quarantine of infected individuals can stop the spread of the infection. This also means that less precise contact tracing will increase the number of susceptible quarantined individuals. In other words, for the same number of traced quarantined people, a less precise tracing will be less effective.

The set of Equations 6 do not have an analytical solution, so we have to use a numerical solution such as the Euler method, or even more efficient algorithms such as the built-in Matlab function ode45 used in this paper. Initially, we assume a number of infected individuals $I(0)$ and recovered individuals $R(0)$, meaning that $S(0)=N-I(0)-R(0)$, and the other classes are zero. Then, the model is solved for a given time (one year, for example) or until the number of infected individuals (the sum of classes $I,Q_{I},Q_{T}$) is less than one. The latter means that the infection has finished and the duration of the epidemic can be obtained as the time when $I(t)+Q_{T}(i)+Q_{I}(t) < 1$.

As in the stochastic model, we can also obtain the accumulated number of individuals that have been quarantined by tracing as:

\begin{equation*} Q_{a} = q_{i}\frac {k(1-b)IS}{N} + q_{s}\frac {kbIS}{N} \tag{8}\end{equation*}

Thus, $Q_{a}(t)$ is the total number of individuals quarantined by tracing up to time $t$.

## V. Evaluation of the Models

This section evaluates the previous models, their precision and applicability. It also shows the dynamics of the epidemic when considering different quarantine measures.

### A. Comparison of Stochastic and Deterministic Models

In order to use the deterministic model, it is necessary to evaluate how the results of both the stochastic and deterministic models match. To evaluate both models, we used the estimated COVID-19 parameters shown in Table 3. In the following experiments, we evaluate the spread of the infection using the NCCU trace, assuming an initial outbreak at day one with five infected individuals ($I(0) = 5$), with no recovered individuals ($R(0) = 0$), and the tracing time was set to one day ($1/\tau _{T}=1$), reflecting fast mobile tracing. For the estimated contact network $\widehat {\mathbf {G}}(t)$, we used the one obtained using Bluetooth technology. For the stochastic model, we performed 30 realisations, selecting the initially infected individuals for each realisation randomly. Using all these realisations, we also obtained the averages of the main curves, i.e., the number of Susceptible, Infected, and Recovered individuals. From these realisations, we also obtained the value of $k,q_{i},q_{s}$ to be used in the deterministic model.

**Figure 3.**
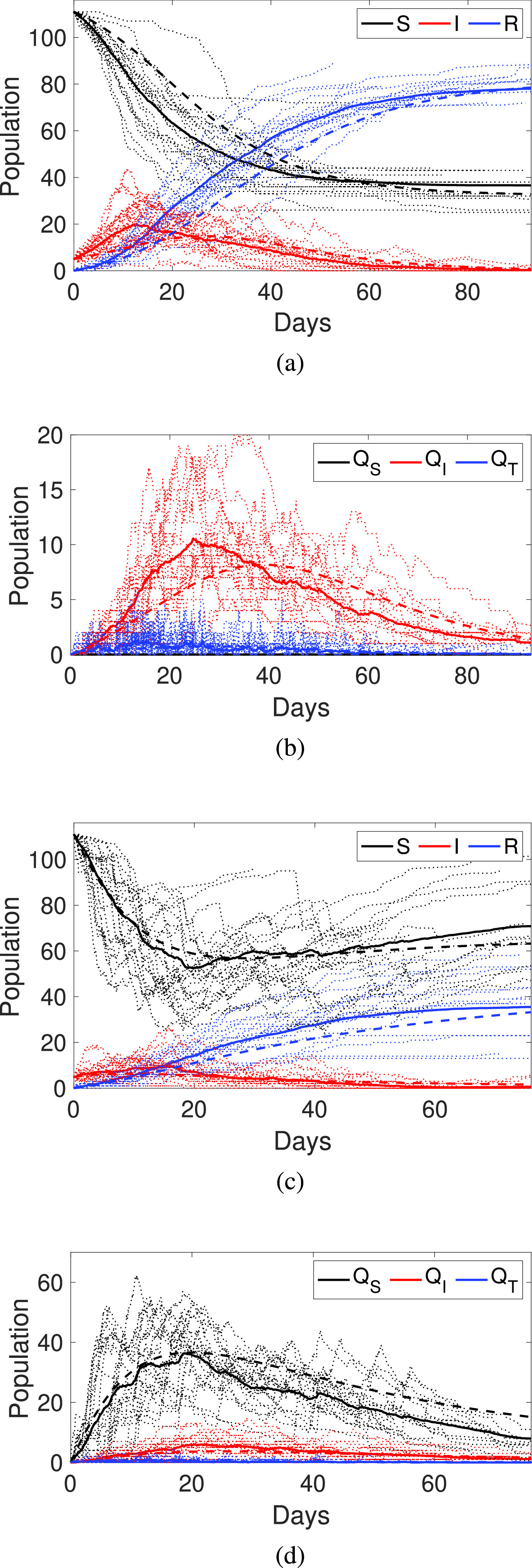
Dynamics of the COVID-19 epidemics in the NCCU trace. a) and b) without tracing quarantine; c) and d) using tracing quarantine with $q=0.6$. Dotted lines are the results of 30 realisations using the stochastic model; Solid lines are the average of these stochastic realisations, and dashed lines are the results using the deterministic model.

The first experiment considers no tracing quarantine measures are taken, that is, when $q=0$. Figure 3b shows the number of individuals in the $S$, $I$ and $R$ states (classes). Considering the average curves of the stochastic model (solid lines), we can see that the number of infected individuals initially increases, but after fifteen days (peak of infections) the infection diminishes, and it ends around the eightieth day. Figure 3b shows the number of individuals quarantined, so we can see the peak of quarantined individuals takes place on about day twenty-five. Regarding the differences between the two models, we can see that, in general, the deterministic model (dashed lines), when compared with the average of the stochastic model (solid lines), slightly overestimates the number of infections.

The second experiment considers a tracing quarantine with $q=0.6$. With this value, the estimated fractions of traced contacts quarantined for the deterministic model are $q_{i}=0.052$ and $q_{s}=0.127$. In this case, we can see in Figure 3c that the number of infected individuals has been reduced when compared to the no-tracing quarantine results but at the cost of increasing the number of individuals quarantined, as shown in Figure 3d. The duration of the infection is slightly shorter, showing the effect of the quarantine. The most significant difference between the average stochastic and deterministic curves is the traced susceptible quarantined ($Q_{S}$). The reason for this difference is that, in the stochastic model, it is more likely for an individual being traced to get infected (she/he has been in contact with an infected person). This implies that transition $S \rightarrow Q_{S}$ is less probable than in the deterministic model, in which all traced nodes are evaluated homogeneously.

**Figure 4.**
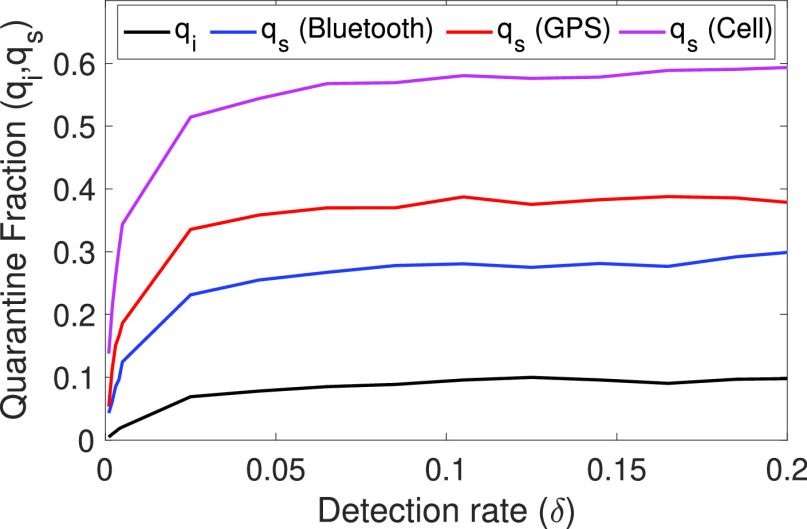
Estimated maximum values for the fraction of tracing quarantine ($q_{i}$, $q_{d}$) to use in the deterministic model depending on the detection ratio $\delta $.

We repeated the previous experiments using other tracing contact networks $\widehat {\mathbf {G}}(t)$ (that is, considering *GPS*, *Wi-Fi* and *Cell* technologies for contact tracing) and the results were quite similar. The effect of the tracing mechanism is measured in the obtained values of $q_{i}$ and $q_{s}$. These values not only depend on $q$, but also on the detection rate $\delta $ (and, of course, the contact network studied). Thus, to use the deterministic model, we can estimate $q_{i}$ and $q_{s}$ for any given $q$ and $\delta $ values. Depending on these values, there is an upper limit range on $q_{i}$ and $q_{s}$, as shown in Figure 4. We can see that due to a lack of precision for detecting true contacts, some technologies can trace up to 60% of the susceptible individuals that have been in contact with infected individuals, meaning most of them would be false positives (individuals traced and quarantined, but not likely to have been infected). Additionally, these results restrain the study of the deterministic model to these limit values. Considering these issues, we can use the deterministic model to evaluate large populations, which is not computationally amenable using the stochastic model.

### B. Epidemic Dynamics

In this subsection, we study the epidemic dynamics using the deterministic model described by Equations 6 depending on the different quarantine strategies. The idea is to provide a better understanding of the dynamics of quarantining using contact tracing. We again use the COVID-19 disease estimated parameters shown in TABLE 3 with a population of $N=1$ million, and an initial number of infected individuals set at 10 ($I(0)=10$), with no immunised individuals ($R(0)=0$) and an average of eight contacts per day ($k=8$).

**Figure 5.**
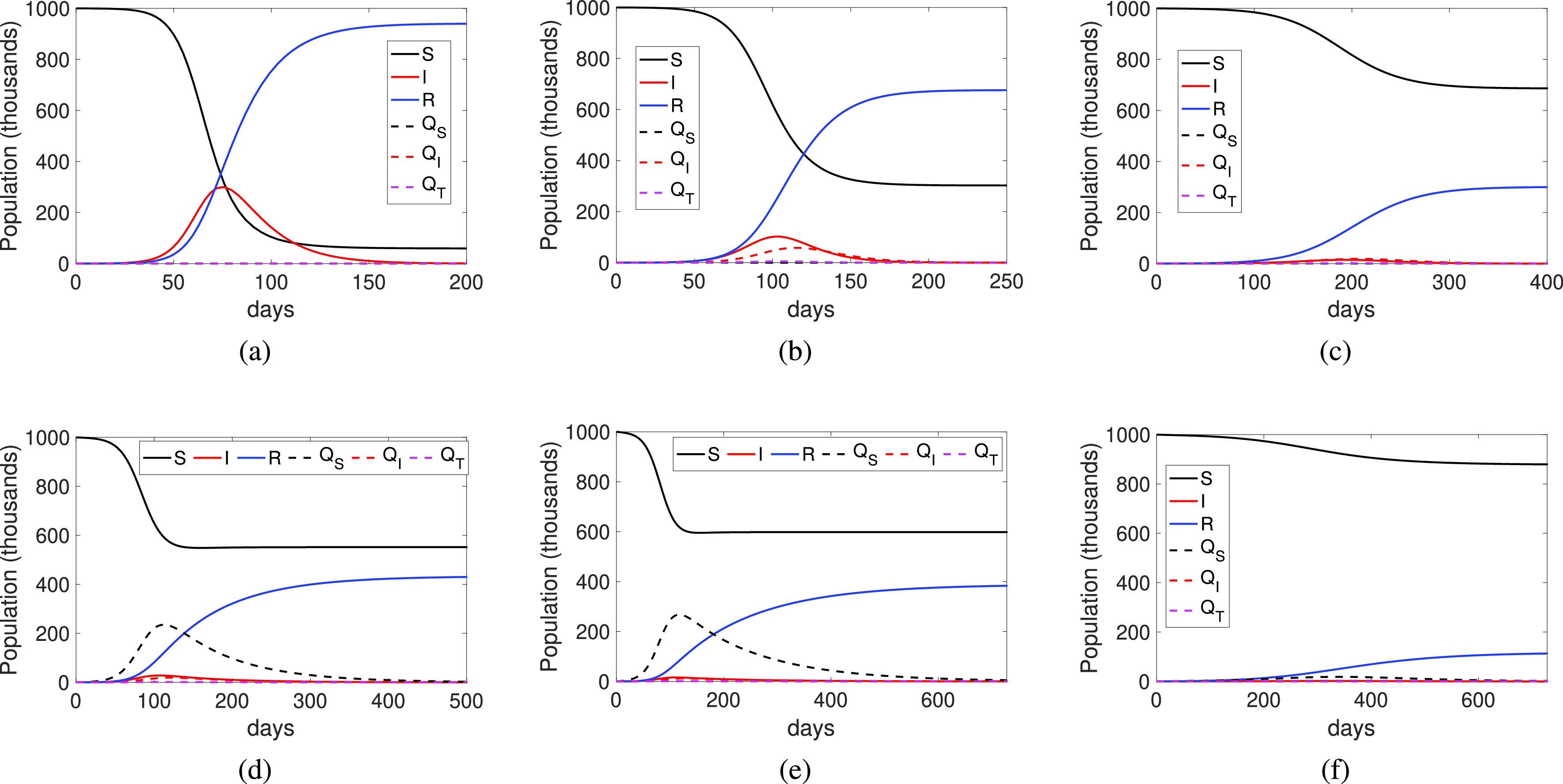
Epidemic Dynamics under different quarantine methods for the COVID-19 infection. a) No measures ($\delta =q=0$); b) Detection and isolation of individuals. ($\delta =0.05$, $q_{i}=q_{s}=0$) c) Increasing detection ratio ($\delta =0.1$, $q_{i}=q_{s}=0$); d) Adding tracing quarantine ($\delta =0.05$, $q_{i}=0.05,q_{s}=0.13$); f) Increasing tracing quarantine ($\delta =0.05$, $q_{i}=0.08,q_{s}=0.25$); g) Increasing also the detection ratio ($\delta =0.1$, $q_{i}=0.1, q_{s}=2.8$).

We start by considering the case when no measures are taken, that is, individuals are neither detected nor quarantined ($\delta =q_{i}=q_{s}=0$). The results are shown in Figure 5a, which clearly represents a typical simple SIR model where most of the population gets infected due to the high reproductive ratio ($R_{0}$). In the second Figure 5b, we consider that some of the infected individuals are detected and isolated (that is, the detection rate is $\delta =0.05$). We can see that this simple measure reduces the number of individuals that get infected, although the duration of the infection is slightly increased due to a small number of remaining infected individuals, which slowly drops off after day 150. If we increase the detection ratio to $\delta =0.1$, for example by increasing the number of tests performed, the infected curve is flattened, as shown in Figure 5c, significantly reducing the final number of infected individuals. Flattening the curve of infected individuals also implies that the duration of the infection increases.

Now, we evaluate the impact of the contact tracing quarantine. Figure 5d shows the result for $q_{i}=0.05$, $q_{s}=0.13$ and $\delta =0.05$. These values correspond to $q=0.6$ in the stochastic model. It can be observed that the number of infected nodes is reduced when compared to the results in Figure 5b, although the duration of the infection is increased. Note also that, as expected, the number of quarantined individuals (dashed lines) has also increased. If we increase $q_{i}, q_{s}$ to the maximum allowed for $\delta =0.05$, which is $q_{i}=0.08,q_{s}=0.25$, the infected population is notably reduced, as shown in Figure 5e, and the quarantined individuals are also reduced. Finally, Figure 5f shows the effect of increasing the detection ratio to $\delta =0.1$ with $q_{i}=0.1$ and $q_{i}=2.8$. In this case, the curve of those infected has been completely flattened, and the number of infected individuals is also reduced. If we increase these values, for example to $q_{i}=0.1$ and $\delta = 0.15$, the result (not shown here since they are flat curves) is a control of the outbreak, and the infection is not spread. These threshold values can be obtained using Equation 7, considering nearly all people to be susceptible $S/N=1$. We will study this issue in detail in the next section.

Summing up, the right selection of the detection rate along with quarantine measures has a huge impact on controlling the spread of the infection. In the next section, we will study in detail the best combination of these measures.

## VI. Efficiency of Quarantine Measures and Tracing Technologies

This section extends the previous experiments to determine the effectiveness of each quarantine measure, and thus the optimal strategy. It also evaluates the precision of the different contact tracing technologies.

### A. Efficiency of Quarantine Measures

**Figure 6.**
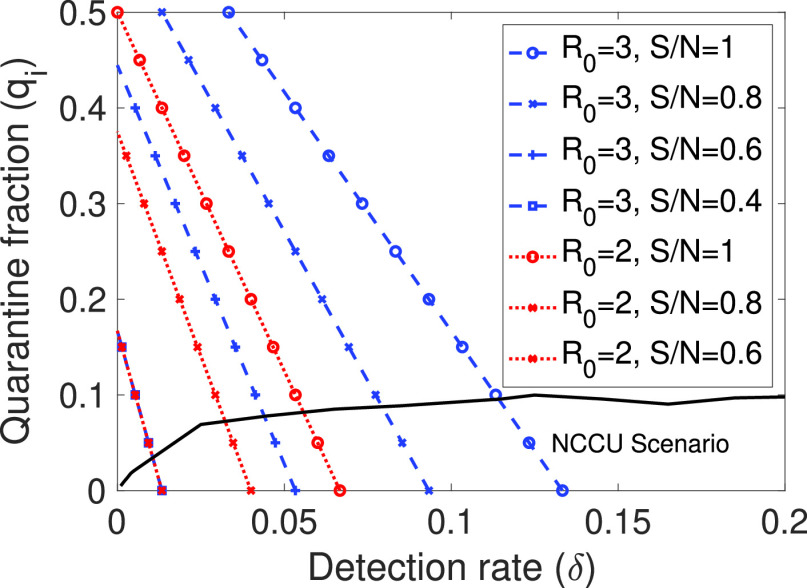
Threshold for infection control for different ratios of susceptible population. The pair of values above the dashed line results in a disease-free equilibrium. The area delimited by the black line corresponds to the possible values for the NCCU scenario.

First, we evaluate the threshold for controlling an outbreak (that is, when the number of infected individuals decreases) using Equation 7, depending on the proportion of susceptible people $S/N$ and the basic reproductive ratio $R_{0}$. We evaluate a first epidemic outbreak for not only when all individuals were susceptible ($S/N=1$), but also when some proportion of the population has gained immunity after having recovered. This fact is a key issue in controlling future and localised waves of infection since some population will be immunised. The results are shown in Figure 6. Note that only the values delimited by the black lines correspond to the possible values for the NCCU scenario.

The presented results are very significant, especially when considering that COVID-19 has an estimated average detection rate of 0.05 and a reproductive ratio $R_{0}$ close to 3. We can see that, for the initial outbreak when $S/N=1$, even using efficient contact tracing, the disease cannot be controlled, as unfortunately has happened. On the contrary, when some fraction of people get immunised, outbreaks can be better controlled using smartphone contact tracing applications. For example, for $S/N=0.8$, we can obtain an outbreak control for $q_{i}=0.05$ and $\delta =0.085$. Note that for the NCCU scenario, a value of $q_{i}=0.05$ corresponds to approximately $q=0.6$, that is, the required fraction of people using the contact tracing application. This means that the previous requirement of having a significant fraction of people using the contact tracing application is relaxed. We can also see that, if $S/N < 0.5$, the quarantine will not be necessary as the system will be close to *herd immunity*. Additionally, if we consider the concurrent application of future measures like relaxed social distancing (after the strict initial lockdowns), which can reduce the reproductive ratio $R_{0}$, we can also see in Figure 6 that for $\delta =0.04$ with relatively small values of $q_{i}$ (between 0.01 and 0.04), the outbreak can be controlled. The consequence is that the required fraction of people that must be using the application is reduced to values of between 0.2 to 0.4, making it a feasible solution.

**Figure 7.**
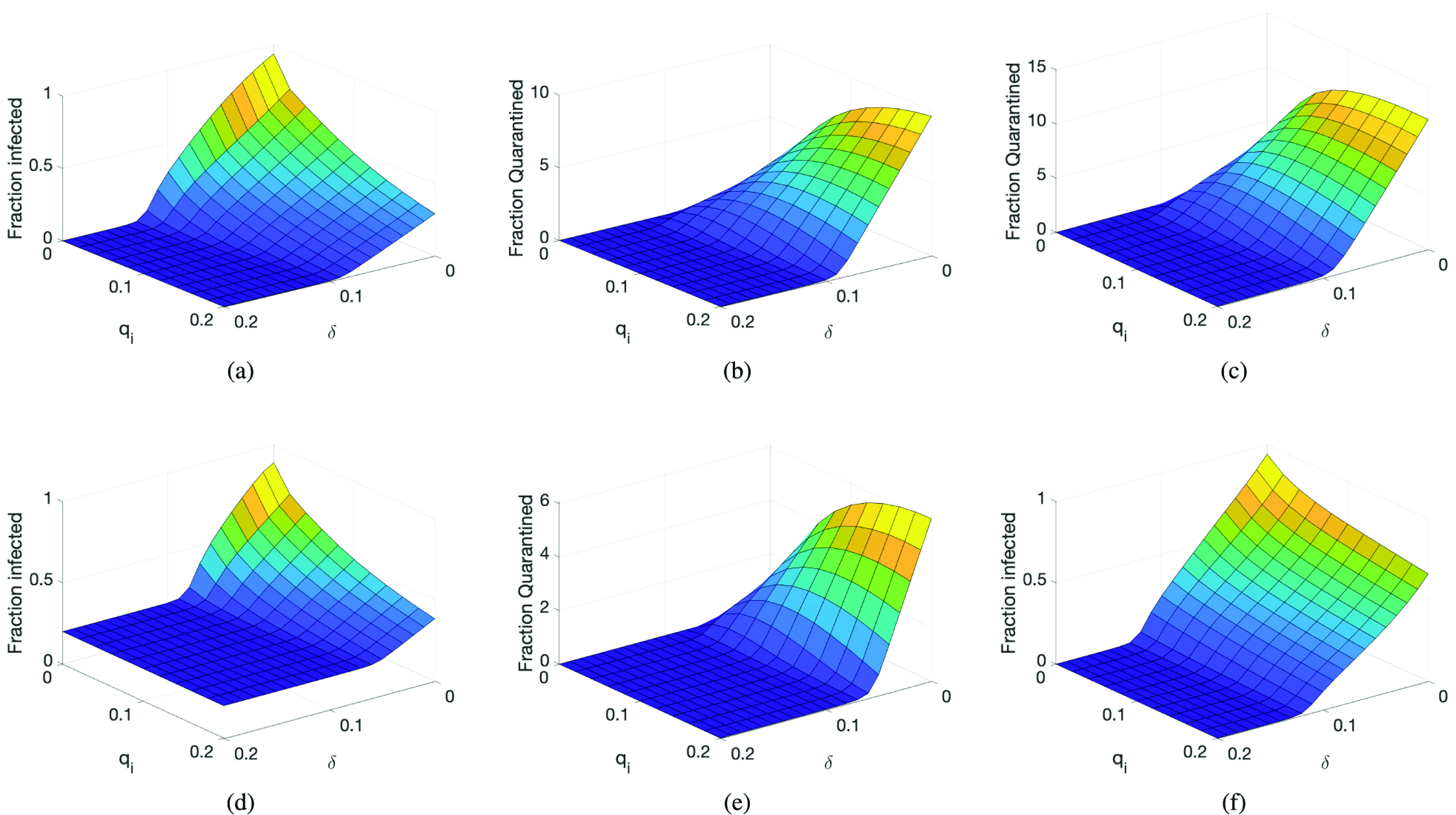
Quarantine measure effectiveness: a) Fraction of population infected using Bluetooth-based tracing contact technology ($R(0)=0$); b) Fraction of population quarantined using Bluetooth-based tracing contact technology ($R(0)=0$); c) Fraction of population quarantined using GPS-based tracing contact technology ($R(0)=0$); d) Fraction of population infected using Bluetooth-based tracing contact technology ($R(0)=0.2N$); e) Fraction of population quarantined using Bluetooth-based tracing contact technology ($R(0)=0.2N$).

We now evaluate the case of when the infection has not been controlled, and thus many individuals get infected. This evaluation is performed using the deterministic model, but only showing the final results, that is, when the infection is over. Again, the same COVID-19 disease parameters are used with a population of 1 million and considering Bluetooth technology for tracing contacts. The infected and quarantine values are expressed as a fraction of the population (i.e. divided by $N$). Figure 7a shows the fraction of the infected population depending on the detection rate ($\delta $), and the fraction of traced contacts quarantined ($q_{i}$). We can clearly see how the infection is reduced when both the detection rate and quarantine measures are considered. We can contrast these results with the fraction of population quarantined, as shown in Figure 7b, where for low values of detection rates, the quarantined population can be very high.^8^ Using a less precise tracing technology (*GPS*), we found that, as expected, the fraction of quarantined people increases, as shown in 7c. Finally, if we consider that part of the population is already immunised, with $R(0)=0.2N, S(0)\approx 0.8N$, the fraction of newly infected individuals is significantly reduced when considering contact tracing, and additionally, the number of quarantined people practically drops to half.^8^We again point out that a fraction of quarantined population greater than one means that individuals have been quarantined more than one time or for longer periods than the quarantine time used in the model.

Summing up, for the first outbreaks of COVID-19, smartphone-based contact tracing can only be effective when using fast and high-precision contact tracing technology and when considering that a significant proportion of the population uses the application. It is also shown that the number of people quarantined by contact tracing can be very high if the detection rate is low, as it is for COVID-19. Fortunately, for future outbreaks, and considering that at least 20% of individuals could be immunised, or that the reproductive ratio is reduced by some mild social distancing measures, smartphone contact tracing applications can be very effective.

### B. Efficiency of Contact Tracing Technology

In this subsection, we evaluate two aspects related to smartphone contact tracing technology: its speed and contact detection precision, and how it impacts the control and spread of diseases.

One of the advantages of using smartphone-based contact tracing is its speed. When detecting an infected individual, tracing back his/her contacts is almost immediate. With traditional contact investigation it can take several days to obtain these prior contacts. Therefore, we evaluated the impact of this tracing time by increasing the value of $1/\tau _{T}$ to 5 days, and repeating the experiment of Figure 7a. The results are shown in Figure 7f, evidencing a substantial increase in the fraction of infected individuals, and confirming how important tracing prior contacts is to having an effective quarantine.

We shall now study the efficiency of the different contact tracing methodologies described in Section III. In previous experiments, we have shown that using a more precise contact network has no impact on reducing the number of infected individuals since this aspect mainly depends on the value of $q_{i}$. However, using imprecise contact networks can increase the number of quarantined individuals, resulting in a similar number of infected individuals. Thus, using a more precise tracing technology will allow for greater selectivity of individuals who are more likely to have been infected, reducing the overall number of quarantined people.

**Figure 8.**
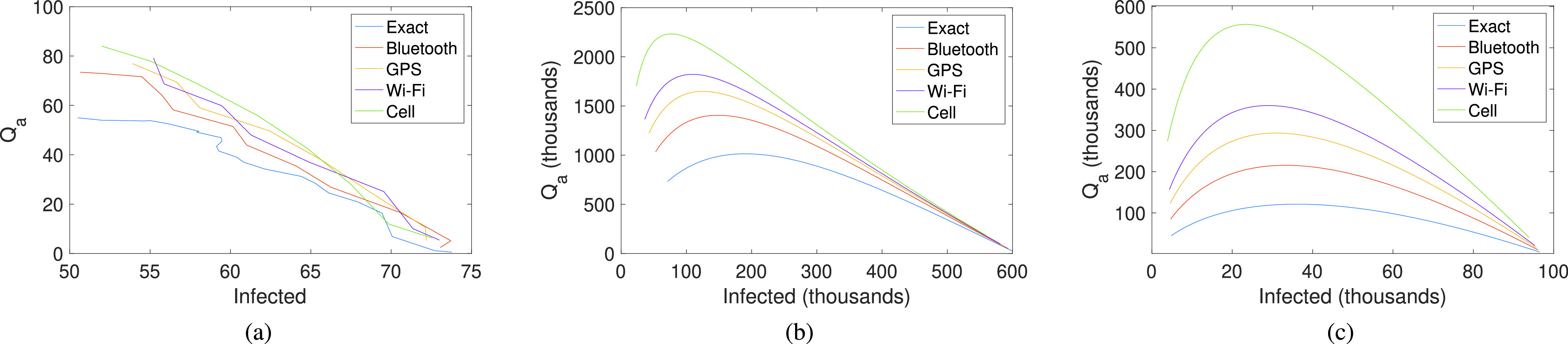
Evaluation of the contact tracing technology considering the final number of infected individual versus the required quarantine effort ($Q_{a}$). a) In the NCCU scenario using the Stochastic model; b) Using the deterministic model for $R(0)=0$; c) Using the deterministic model for $R(0)=0.2N$. Note that, in this case, infected people corresponds to the newly infected (not considering the previous ones).

To confirm this, we performed the following experiment using the stochastic model and the NCCU mobility trace. The idea was to obtain the accumulated number of quarantined individuals ($Q_{a}$) versus the final number of infected individuals. This way, we can compare the quarantine efforts required for each contact tracing technology. The results are shown in Figure 8a, which shows the average results of performing 500 stochastic realisations. We can clearly see that the more precise the technology is, the fewer people that get quarantined, with no increase in the number of infected individuals.

Using the values of $q_{i}$ and $q_{s}$ obtained from this experiment, we can evaluate the effect of tracing technology over a more extensive population using the deterministic model. For this experiment, we again consider, as in Subsection V-B, a population of $N=1$ million, and an initial number of infected individuals of 10 ($I(0)=10$), with no immunised individuals ($R(0)=0$), and an average of eight contacts per day ($k=8$). The results are shown in Figure 8b, which give a pattern similar to the results for the stochastic model. Finally, we evaluate the case when there is a fraction $R(0)=0.2N$ of the population already infected (that is, considering possible new outbreaks at second waves). The results (see Figure 8c) show that the number of quarantined citizens is reduced and that the number of newly infected individuals can be controlled with low quarantine efforts when highly accurate tracing technologies such as Bluetooth are adopted.

Summing up, as expected, mobile contact tracing technology exhibits a huge impact on the quarantine of individuals. Firstly, it is absolutely vital for it to be fast. Second, it must be precise when detecting real contacts.

## VII. Conclusion

In general, accurate modelling and quantifying of human mobility is critical to improving control of epidemics, but it may be hindered by data incompleteness or unavailability [18]. Furthermore, depending on the technology used, we can obtain varying accuracy on the contact tracing.

In this paper, we focused on evaluating how smartphone contact tracing technology can impact the control and spread of infectious diseases. We have introduced a stochastic model that gets transformed into a deterministic model, while taking into consideration the effect of contact tracing and the quarantine measures. On the basis of these models, we evaluated and compared several possible scenarios for smartphone-based contact tracing. Although these models are generic for infectious diseases, we have studied the case of COVID-19 in particular.

With regard to contact tracing technology, our results show that the mobile technology used for detecting contacts has a great impact on the social and economic cost (measured as the number of people quarantined). Accurate technologies, such as Bluetooth, allow for greater selectivity when it comes to quarantining people.

Furthermore, the results also show that in order to be effective (and particularly for the COVID-19 infection), mobile contact tracing requires contacts be traced quickly and a very high percentage of the population must use the contact tracing App. Fortunately, our study also shows that for possible second waves of infection, mobile contact tracing can be effective in controlling the disease, assuming that some portion of the population will have gained immunity, or in combination with some other lenient measures, such as social distancing.
